# Ultrasonic Flaw Echo Enhancement Based on Empirical Mode Decomposition

**DOI:** 10.3390/s19020236

**Published:** 2019-01-09

**Authors:** Wei Feng, Xiaojun Zhou, Xiang Zeng, Chenlong Yang

**Affiliations:** 1 State Key Lab of Fluid Power and Mechatronic Systems, Zhejiang University, Hangzhou 310027, China; fengweizju@126.com (W.F.); cmeesky@163.com (X.Z.); 2 CRRC Zhuzhou Institute Co. Ltd., Zhuzhou 412001, China; zzjjuu0104@163.com

**Keywords:** ultrasonic flaw echo enhancement, empirical mode decomposition, sample entropy, Otsu’s method for thresholding, flaw echo separation

## Abstract

The detection of flaw echoes in backscattered signals in ultrasonic nondestructive testing can be challenging due to the existence of backscattering noise and electronic noise. In this article, an empirical mode decomposition (EMD) methodology is proposed for flaw echo enhancement. The backscattered signal was first decomposed into several intrinsic mode functions (IMFs) using EMD or ensemble EMD (EEMD). The sample entropies (SampEn) of all IMFs were used to select the relevant modes. Otsu’s method was used for interval thresholding of the first relevant mode, and a window was used to separate the flaw echoes in the relevant modes. The flaw echo was reconstructed by adding the residue and the separated flaw echoes. The established methodology was successfully employed for simulated signal and experimental signal processing. For the simulated signals, an improvement of 9.42 dB in the signal-to-noise ratio (SNR) and an improvement of 0.0099 in the modified correlation coefficient (MCC) were achieved. For experimental signals obtained from two cracks at different depths, the flaw echoes were also significantly enhanced.

## 1. Introduction

The ultrasonic technique has been widely used in nondestructive testing. Usually, the backscattered signal is complex due to the existence of electronic noise and backscattering noise. Consequently, flaw echo detection may be challenging. Numerous methods have been proposed to enhance flaw echoes, such as split spectrum processing [1,2,3,4], wavelet transforms [5,6,7,8,9,10], the Stockwell transform [11,12,13,14], and empirical mode decomposition (EMD) [15,16,17,18,19,20,21,22] (including the so-called ensemble EMD, i.e., EEMD [23]). 

Split spectrum processing has significant advantages in processing ultrasonic signals with scattered noise. Split-spectrum analysis separates the spectrum of the signals to obtain several sub-bands, and uses some nonlinear de-noising criteria (such as thresholding method, etc.) to process the signals in each sub-band to achieve the purpose of de-noising. The difficulty of split spectrum processing is how to determine the filter type, central frequency, bandwidth and other parameters. In addition, split spectrum processing lacks the capability of multiresolution analysis.

Wavelet transform is a classical multiresolution analysis method. The difficulty of wavelet transform is how to choose the appropriate wavelet base function and decomposition layer. The disadvantage of conventional wavelet transform is that the phase information of signals is lost.

S transform is the development of short-time Fourier transform and wavelet transform. S transform combines the multiresolution analysis ability of wavelet transform and the phase retention ability of short-time Fourier transform. Meanwhile, the S transform adopts Gaussian window function, which satisfies the normalization characteristic, so the S transform is invertible, that is, the original signal can be obtained from the converted time spectrum. However, due to the fact that the standard deviation of Gaussian window function in S transform is inversely proportional to the frequency and lacks flexibility, S transform may output the result of poor time-frequency resolution. At present, to compensate for the limitations of S transform, researchers introduce additional parameters to control the window function morphology, so that the generalized S transform has the ability to flexibly adjust the time-frequency resolution.

For a single component signal, Hilbert transform can be applied to obtain its analytical signal, and then the envelope spectrum and instantaneous frequency of the signal can be obtained. Detection of a flaw signal from the envelope spectrum is a common method of ultrasonic nondestructive testing. For a multi-component signal, it is necessary to decompose them into single component signals, and then obtain their analytical signals separately. Empirical mode decomposition (EMD) is an adaptive decomposition method. The original signal is decomposed into a series of intrinsic mode functions. The main disadvantages of EMD are the possibility of endpoint effect and mode mixing. Ensemble empirical mode decomposition (EEMD) can solve the mode mixing issue. EEMD decomposes the original signal by adding Gaussian white noise, and takes the result of the lumped average as the mode function. The main difficulty of EEMD is to select the intensity of Gaussian white noise and the times of lumped average.

In this article, an EMD-based methodology for ultrasonic flaw echo enhancement was established. The proposed methodology enhanced the flaw echo through six steps. First, the backscattered signal was adaptively decomposed into several intrinsic mode functions (IMFs) by EMD or EEMD. Second, the sample entropies (SampEn) [24,25] of all IMFs were calculated, and the differences in consecutive SampEn values were studied. Third, those IMFs with a large SampEn were considered to be irrelevant modes and were discarded, significantly suppressing the electronic noise. Fourth, the intervals containing the flaw echo were determined based on IMF interval thresholding and mode cell merging. Otsu’s method [26] was used to search the threshold in IMF interval thresholding. Fifth, a Turkey-Hanning window was used to separate the flaw echo for each relevant mode. Finally, the denoised signal was reconstructed by combining the separated flaw echoes and the residue.

The remainder of this article is organized as follows. In Section 2, reviews of the required tools, including EMD and EEMD, EMD-based denoising methods, SampEn, and Otsu’s method for thresholding, are presented. An analysis of the modes extracted from the backscattered signal, including the mixing of noise and flaw echoes, and the SampEn is given in Section 3. The proposed EMD-based methodology for ultrasonic flaw echo enhancement is given in Section 4 and tested using a simulated signal in Section 5. In Section 6, experimental validations of the proposed EEMD-based methodology are presented. Finally, conclusions are drawn in Section 7.

## 2. Required Tools

### 2.1. EMD and EEMD

In EMD, a signal is adaptively decomposed into a collection of IMFs. The EMD results can be presented as

(1) $$x(t)=\sum_{i=1}^{L}h^{\left(i\right)}(t)+r(t)$$

where x(t) is the observed signal, $h^{\left(i\right)}(t)(i\leq L)$ are the extracted IMFs, and r(t) is the residue.

Unfortunately, EMD is susceptible to mode-mixing. EEMD is an effective technique for alleviating mode-mixing in EMD by repeatedly adding Gaussian white noise and finding the mean of the individual ensemble IMFs as the final IMF.

### 2.2. Denoising Strategies Based on EMD

Partial reconstruction, direct thresholding, and interval thresholding are three typical strategies adopted in EMD-based denoising. 

Partial reconstruction removes noise from the observed signal by discarding irrelevant modes, which can be expressed as

(2) $$\hat{x}(t)=\sum_{i=M_{1}}^{L}h^{\left(i\right)}(t)+r(t)=x(t)-\sum_{i=1}^{M_{1}-1}h^{\left(i\right)}(t)$$

where $h^{\left(i\right)}(t)(i<M_{1})$ are the irrelevant modes.

Direct thresholding is a direct application of wavelet thresholding in the EMD case. For the hard thresholding case, the denoised IMF is given by

(3) $${\tilde{h}}^{\left(i\right)}(t)=\{\begin{array}{ll} h^{\left(i\right)}(t), & \left|h^{\left(i\right)}(t)\right|>T_{i}\\ 0, & \left|h^{\left(i\right)}(t)\right|\leq T_{i} \end{array}$$

where $T_{i}$ is the threshold of $h^{\left(i\right)}(t)$. The denoised signal can be given by

(4) $$\tilde{x}(t)=\sum_{i=M_{1}}^{M_{2}}{\tilde{h}}^{\left(i\right)}(t)+\sum_{i=M_{2}+1}^{L}h^{\left(i\right)}(t)+r(t)$$

The interval thresholding divides an IMF into several mode cells and treats each mode cell as a whole to perform thresholding. Generally, a mode cell is defined as the signal between two adjacent zero-crossings. For the interval $z_{j}^{\left(i\right)}=[z_{j}^{\left(i\right)}z_{j+1}^{\left(i\right)}]$ defined by two zero-crossings $z_{j}^{\left(i\right)}$ and $z_{j+1}^{\left(i\right)}$, the denoised IMF in the hard thresholding case is given as

(5) $${\tilde{h}}^{\left(i\right)}(z_{j}^{\left(i\right)})=\{\begin{array}{ll} h^{\left(i\right)}(z_{j}^{\left(i\right)}), & \left|h^{\left(i\right)}(r_{j}^{\left(i\right)})\right|>T_{i}\\ 0, & \left|h^{\left(i\right)}(r_{j}^{\left(i\right)})\right|\leq T_{i} \end{array}$$

where $h^{\left(i\right)}(z_{j}^{\left(i\right)})$ are all of the samples from $z_{j}^{\left(i\right)}$ to $z_{j+1}^{\left(i\right)}$, and $h^{\left(i\right)}(r_{j}^{\left(i\right)})$ is the single extremum of $h^{\left(i\right)}(t)$ in the interval $z_{j}^{\left(i\right)}$.

Interval thresholding generally outperforms direct thresholding as it avoids catastrophic consequences for the continuity of the reconstructed signal, which are inevitable in direct thresholding.

### 2.3. Sample Entropy

Approximate entropy [27] and SampEn are two popular metrics for signal complexity measurement. Entropy values increase with increased signal complexity. It has been reported that SampEn outperforms approximate entropy in many aspects, such as reduced bias, independence from the signal, and relative consistency. SampEn was used here for signal complexity assessment.

The SampEn of a specified time series {u(i),i=1,2,⋯,N} can be obtained through the following steps.

Step 1: Form m−dimensional vectors as

(6) $$U_{m}(i)=\left[u(i),u(i+1),\ldots ,u(i+m-1)\right]$$

where i=1,2,…,N−m+1.

Step 2: Define the distance of two such vectors:

(7) $$d\left[U_{m}(i),U_{m}(j)\right]=\underset{k=0-(m-1)}{\max}\left|u(i+k)-u(j+k)\right|$$

Step 3: Consider the first N−m vectors of length m so that for i=1,⋯,N−m, both $U_{m}(i)$ and $U_{m+1}(i)$ can be defined.

Step 4: Given a threshold r>0, define

(8) $$\begin{array}{l} B_{i}^{m}(r)=\frac{1}{N-m-1}\sum \Theta (r-d\left[U_{m}(i),U_{m}(j)\right])\\ A_{i}^{m}(r)=\frac{1}{N-m-1}\sum \Theta (r-d\left[U_{m+1}(i),U_{m+1}(j)\right]) \end{array}$$

where j=1,2,…,N−m,j≠i, and Θ(·) is defined as

(9) $$\Theta (x)=\{\begin{array}{ll} 1, & x\geq 0\\ 0, & x<0 \end{array}$$

Step 5: Calculate $B^{m}(r)$ and $A^{m}(r)$:

(10) $$\begin{array}{l} B^{m}(r)=\frac{1}{N-m}\sum_{i=1}^{N-m}B_{i}^{m}(r)\\ A^{m}(r)=\frac{1}{N-m}\sum_{i=1}^{N-m}A_{i}^{m}(r) \end{array}$$

Step 6: For a limited series, the SampEn (denoted by s) is estimated as

(11) $$s=SampEn(m,r)=-\ln\frac{A^{m}(r)}{B^{m}(r)}$$

In general, the dimension and threshold are often set to m=2 and $r=(0.1∼0.25)SD_{u}$, where $SD_{u}$ is the standard deviation of the time series {u(i)}.

### 2.4. Otsu’s Method for Thresholding

Otsu’s method determines a threshold by maximizing the between-class variance $\sigma_{B}^{2}$. For a histogram with H levels (i.e., bins), the probability at each level can be first obtained:

(12) $$p_{i}=\frac{n_{i}}{N},i=1,2,\cdots ,H$$

where $n_{i}$ is the number of elements in the $i_{\mathrm{th}}$ level, and N is the number of elements in the histogram. Obviously, $p_{i}\geq 0$ and $\sum p_{i}=1$ are satisfied.

The histogram can be divided into two classes, $C_{1}$ and $C_{2}$, with a threshold. $\sigma_{B}^{2}$ is defined as

(13) $$\sigma_{B}^{2}=\omega_{1}{\left(\mu_{1}-\mu_{0}\right)}^{2}+\omega_{2}{\left(\mu_{2}-\mu_{0}\right)}^{2}$$

where

(14) $$\begin{array}{ccc} \omega_{1}=\sum_{C_{1}}p_{i}, & \omega_{2}=\sum_{C_{2}}p_{i}, & \omega_{1}+\omega_{2}=1 \end{array}$$

$\mu_{0},\mu_{1},\mu_{2}$ are the means of the histogram, class $C_{1}$ and class $C_{2}$, respectively. Therefore, Equation (15) can be obtained:

(15) $$\mu_{0}=\omega_{1}\mu_{1}+\omega_{2}\mu_{2}$$

According to Equations (13)–(15), $\sigma_{B}^{2}$ can also be expressed as

(16) $$\sigma_{B}^{2}=\omega_{1}\omega_{2}{\left(\mu_{1}-\mu_{2}\right)}^{2}$$

## 3. Analysis of Modes from Ultrasonic Signals

### 3.1. The Clutter Model

For metallic materials, when an incident ultrasonic wave propagates into the specimen, the backscattered signal will be primarily composed of three components: (1) the flaw echo signal s(t), (2) backscattering noise v(t) due to the grains, and (3) electronic noise n(t) due to the instruments and the environment. n(t) can be approximated as Gaussian white noise. The frequency spectra of s(t) and v(t) can be expressed as [2]

(17) $$V(\omega )=H_{t}{}^{2}(\omega )\sum_{k=1}^{K}\beta_{k}\frac{\omega^{2}}{x_{k}}e^{-\alpha_{s}2x_{k}\omega^{4}}e^{-i\omega \frac{2x_{k}}{c_{0}}}$$

(18) $$S(\omega )=H_{t}{}^{2}(\omega )\exp(-\alpha_{s}2d_{\mathrm{flaw}}\omega )\exp(-i\frac{2d_{\mathrm{flaw}}}{c_{0}})$$

where $H_{t}(\omega )$ is the frequency response of the ultrasonic transducer, and $d_{\mathrm{flaw}}$ is the location of the flaw. $\alpha_{s}$ is the material attenuation coefficient, $c_{0}$ is the velocity of the longitudinal waves, and K is the total number of scatterers. $\beta_{k}$ and $x_{k}$ are the scattering coefficient and the position of the $k_{\mathrm{th}}$ scatterer, respectively. The amplitudes of s(t) and v(t) are often normalized for brevity.

The similarity function obtained by deconvolution can be used to distinguish the flaw signals in ultrasonic inspection from other no flaw signals, such as specimen geometric reflection. It was found that the deconvolution patterns of the geometric reflection were impulse-like patterns, whereas those of flaws were bipolar patterns [28]. Therefore, geometric reflection was not considered in the model.

The observed backscattered signal x(t) can be expressed as

(19) $$x(t)=s(t)+\mu v(t)+\sigma_{n}n_{0}(t)$$

where $n_{0}(t)$ is standard Gaussian white noise. μ and $\sigma_{n}$ are scale factors.

**Figure 1:**
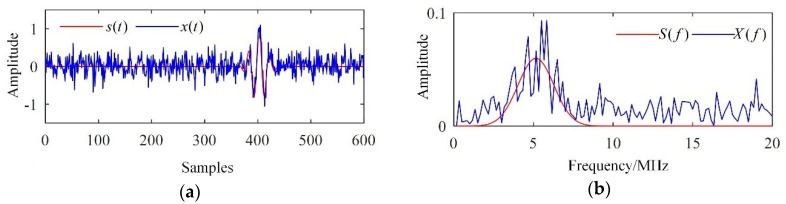
Simulated results. (**a**) Waveforms and (**b**) frequency spectra.

Typical simulated results are depicted in Figure 1. The centre frequency of the ultrasonic transducer was 5 MHz, and the sampling frequency was 100 MHz. The scale factors were set to μ=0.3 and $\sigma_{n}=0.2$. It can be seen in Figure 1a that the flaw echo had been polluted by intense noise. In addition, frequency aliasing of the flaw echo, backscattering noise, and wide-band Gaussian white noise can be found in Figure 1b.

### 3.2. Signal Decomposition

**Figure 2:**
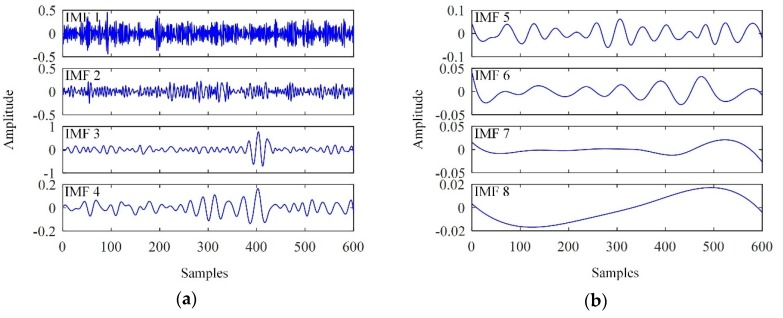
Intrinsic mode functions (IMFs) extracted. (**a**) IMF 1 ~ IMF 4; (**b**) IMF 5 ~ IMF 8.

The EMD results for the observed signal x(t) are shown in Figure 2.

As shown in Figure 2, intense white noise was found in the low-order IMFs; in particular, IMF 1 resembled pure noise. The flaw echo was clearly detected in IMF 3. Consequently, the flaw echo was significantly enhanced by discarding IMF 1 and IMF 2 from the observed signal. However, partial reconstruction is often inadequate; further processing is required. Here, we take IMF 3 as an example.

The flaw echo is an instant signal. Ideally, oscillations can only be detected in the interval in which the flaw echo is located in IMF 3, in contrast to Figure 2. The difference mainly arises from frequency aliasing of the flaw echo, backscattering noise and wide-band Gaussian white noise. In addition, even in the noiseless case, the IMFs still contain false oscillations, as they resemble AM-FM modulated sinusoids. Consequently, further denoising is required to suppress the mixed noise lying outside the interval in which the flaw echo is located.

### 3.3. SampEn Values of IMFs

The SampEn values of all of the IMFs were calculated and are listed in Table 1, where the threshold r in the SampEn calculations was set to $(0.1,0.15,0.2)SD_{u}$.

It can be noted that the SampEn tends to decrease with increasing IMF order, which indicates that the noise intensity in each IMF decreases as IMF order increases. It is noteworthy that the SampEn values of the first two IMFs, i.e., $s_{1}$ and $s_{2}$, were much higher than the others. In addition, a sharp drop between $s_{2}$ and $s_{3}$ was detected.

## 4. Proposed Methodology

According to Section 3.2, flaw echo enhancement can be achieved by discarding irrelevant modes and suppressing the mixed noise in the remaining relevant modes. Specifically, the relevant modes are determined by the SampEn of all IMFs or their differences, and the mixed noise is suppressed by separating the flaw echo from those relevant modes by windowing.

### 4.1. Relevant Mode Selection

The IMFs with intense noise were of much higher complexity than other IMFs. Consequently, those relevant modes were determined according to the SampEn of the IMFs. Specifically, the parameter $M_{1}$, which determined the first relevant mode, was determined by $s_{i}$ or the difference of $s_{i}$.

Given a predefined SampEn threshold $T_{s}$, $M_{1}$ can be determined as

(20) $$\begin{array}{cc} M_{1}=(\max i)+1, & s.t.\;s_{i}>T_{s} \end{array}$$

Using the difference of $s_{i}$, i.e., $ds_{i}$, $M_{1}$ can also be determined:

(21) $$M_{1}=\underset{i}{\arg\min}\{ds_{i}\}=\underset{i}{\arg\min}\{s_{i}-s_{i-1}\}$$

To determine $T_{s}$, we should note that the SampEn is dependent on the threshold r. For example, Table 1 shows that $s_{1}$ and $s_{2}$ decreased rapidly with increasing r. Consequently, the selection of $T_{s}$ was dependent on r. For this article, $r=0.15SD_{u}$ and $T_{s}=1$ were selected.

### 4.2. Mixed Noise Suppression

Two steps are required to suppress mixed noise: determine the location of the flaw echo in the first relevant mode and separate the flaw echo from all of the relevant modes.

For the first relevant mode, i.e., $h^{\left(M_{1}\right)}(t)$, a collection of mode cells is built according to the zero-crossings. The set of absolute values of the extrema in $h^{\left(M_{1}\right)}(t)$, which is denoted $\left|r_{j}^{\left(i\right)}\right|=[\left|r_{1}^{\left(i\right)}\right|,\left|r_{2}^{\left(i\right)}\right|,\cdots ]$, can be determined. Otsu’s method is used for searching the threshold of $\left|r_{j}^{\left(i\right)}\right|$, i.e., $T_{i}$. It is performed on the first relevant mode, and all of the mode cells in which the flaw echoes are located can be detected. 

All of the adjacent mode cells in which the flaw echo is located are further merged, yielding an interval in which the flaw echo is located. For example, three adjacent mode cells, $z_{j}^{\left(i\right)}=[z_{j}^{\left(i\right)}z_{j+1}^{\left(i\right)}]$, $z_{j+1}^{\left(i\right)}=[z_{j+1}^{\left(i\right)}z_{j+2}^{\left(i\right)}]$ and $z_{j+2}^{\left(i\right)}=[z_{j+2}^{\left(i\right)}z_{j+3}^{\left(i\right)}]$, can be merged into one interval $\left[z_{j}^{\left(i\right)}z_{j+3}^{\left(i\right)}\right]$. In other words, the interval in which the flaw echo is located is often composed of several mode cells containing the flaw echo.

Thus far, the location of the flaw echo in the first relevant mode has been detected. Next, the flaw echoes in all relevant modes will be separated.

**Figure 3:**
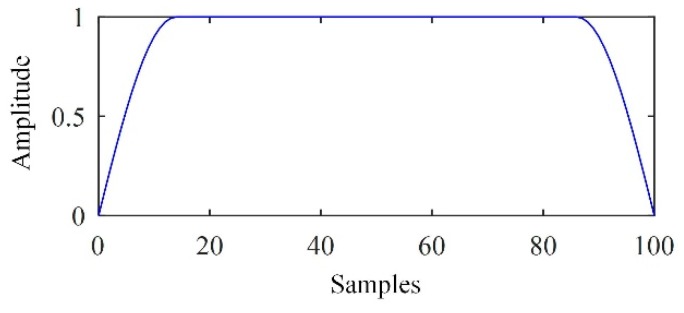
Waveform of a Turkey-Hanning window.

A window was determined and used for separating the flaw echoes in the relevant modes. Herein, the Turkey-Hanning window was used. The window was defined as

(22) $$\omega (m)=\{\begin{array}{ll} \frac{\left(1-\cos2\pi \frac{m}{M+1}\right)}{2}, & 1\leq m\leq \frac{M}{2}\\ 1, & \frac{M}{2}<m\leq \frac{M}{2}+W\\ \frac{1-\cos2\pi \frac{m-W}{M+1}}{2}, & \frac{M}{2}+W<m\leq M+W \end{array}$$

where W determined the width of the pass zone and M determined the width of the transition zone. As an example, Figure 3 shows the waveform of a Turkey-Hanning window.

The parameters W and M were determined according to the width of the interval in which the flaw echo was located. To preserve the flaw echo, W was set to the width of this interval. M was selected more flexibly. In this article, M=[W/4] was adopted, where the operator “[]” indicated rounding.

### 4.3. Summarization of the Proposed Methodology

In this methodology, the flaw echo was enhanced in six steps.

Step 1. Signal decomposition. Decompose the observed signal x(t) into a collection of the residue r(t) and the IMFs’ $h^{\left(i\right)}(t)(i=1,2,\cdots ,L)$ using EMD or EEMD.

Step 2. SampEn calculation. Obtain the SampEn of all of the IMFs.

Step 3. Relevant mode selection. Determine the first relevant mode using the SampEn or the differences between them. 

Step 4. Determine the interval in which the flaw echo is located in the first relevant mode. Otsu’s method is used for threshold selection. Any two adjacent mode cells in which the flaw echo is located are merged into one interval.

Step 5. Separate the flaw echo using the Turkey-Hanning window in each relevant mode, which yields a collection of denoised modes ${\tilde{h}}^{\left(i\right)}(t)$ ($M_{1}\leq i\leq L$). 

Step 6. Reconstruct the denoised signal:

(23) $$\tilde{x}(t)=\sum_{i=M_{1}}^{L}{\tilde{h}}^{\left(i\right)}(t)+r(t)$$

## 5. Simulated Signal Processing

### 5.1. Performance Assessment

To assess the performance of the EMD-based methodology, two metrics are introduced.

The signal-to-noise ratio (SNR) is the first metric. Suppose that s(t) is a noiseless signal; the SNR of signal x(t) is then defined as

(24) $$SNR=10\mathrm{lg}\frac{\sum_{i=1}^{N}s^{2}(i)}{\sum_{i=1}^{N}{\left(x(i)-s(i)\right)}^{2}}$$

Well-preserved flaw echoes are expected in practice. Specifically, the amplitudes and shapes of flaw echoes are expected to be unchanged. This can be assessed by the modified correlation coefficient (MCC), which is defined as

(25) $$MCC=\left|\frac{A_{x}-A_{s}}{A_{s}}\right|(1-\frac{\sum_{i=m}^{n}\left(s(i)-\bar{s})(x(i)-\bar{x}\right)}{\sqrt{\sum_{i=m}^{n}{\left(s(i)-\bar{s}\right)}^{2}\sum_{i=m}^{n}{\left(x(i)-\bar{x}\right)}^{2}}})$$

where m and n are instants defining the interval in which the flaw echo is located. $A_{x}$ and $A_{s}$ are the amplitudes of the flaw echoes in x(t) and s(t), respectively.

A high SNR and low MCC are expected.

### 5.2. Signal Processing Results

**Figure 4:**
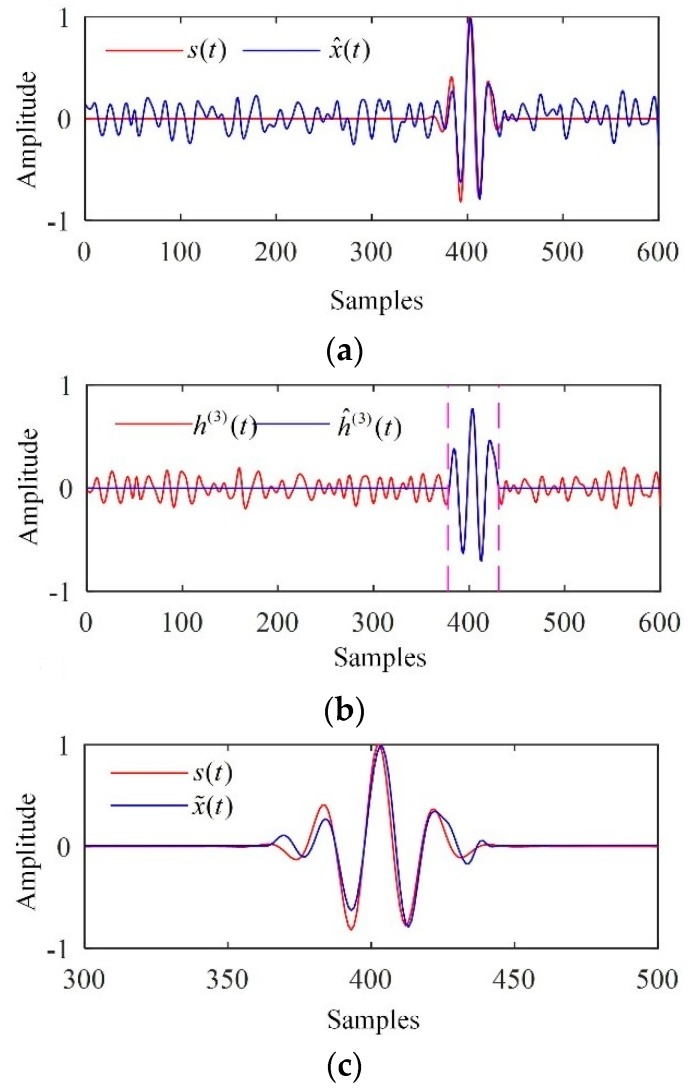
Processing results of the simulated signal. (**a**) The reconstructed signal given by partial reconstruction; (**b**) the detection interval in which the flaw echo was located; (**c**) the denoised signal.

The relevant modes were first determined. On one hand, with the predefined parameters $r=0.15SD_{u}$ and $T_{s}=1$, it can be seen in Table 1 that $s_{1},s_{2}>T_{s}$ and $s_{3}<T_{s}$ and were satisfied. Consequently, $M_{1}=3$ was determined according to Equation (20). On the other hand, it could be inferred from Table 1 that $ds_{3}=s_{3}-s_{2}<ds_{k}(k\neq 3)$ was satisfied, which also indicates $M_{1}=3$ according to Equation (21). Thus, these two methods yielded the same relevant mode selection results. Consequently, the electronic noise could be significantly suppressed if we reconstructed the flaw echo via partial reconstruction by discarding $h^{\left(1\right)}(t)$ and $h^{\left(2\right)}(t)$. The corresponding reconstructed signal, $\hat{x}(t)$, is depicted in Figure 4a. 

$h^{\left(3\right)}(t)$ was used to determine the interval in which the flaw echo was located. The corresponding result is depicted in Figure 4b, for which the threshold given by Otsu’s method was 0.2902. The flaw echoes in all relevant modes $h^{\left(i\right)}(t)$ (3≤i≤8) were separated by the Turkey-Hanning window, and the denoised signal was reconstructed. The denoised signal is depicted in Figure 4c, which indicated that the electronic noise and backscattering noise were significantly suppressed, and the flaw echo were preserved well.

The performance of the EMD-based methodology was assessed using the SNR and the MCC. The results are provided in Table 2, which shows that the methodology achieved a high SNR and low MCC as expected. The improvements in SNR and MCC were 12.89 dB and 0.0099, respectively.

## 6. Experimental Study

**Figure 5:**
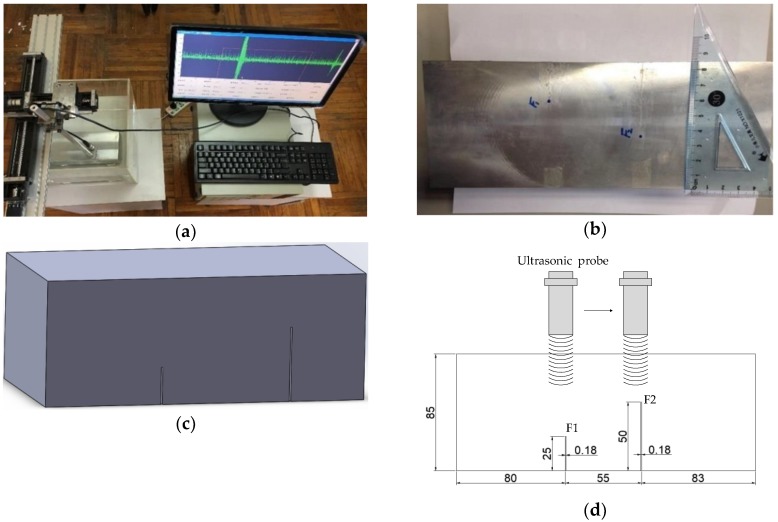
The ultrasonic test system and specimen. (**a**) The ultrasonic testing system. (**b**) The specimen. (**c**) Geometry of the specimen. (**d**) Schematic diagram of flaws location and size, with dimensions in millimeters.

The ultrasonic testing system was primarily composed of an ADVANTECH industrial personal computer (IPC), an Olympus ultrasonic probe with a centre frequency of 5 MHz, and a PCIUT3100 ultrasonic acquisition card installed on the IPC. A 6061 aluminium alloy specimen with two artificial cracks was used for ultrasonic testing. The specimen material density was 2.7 × 10^3^ kg/m^3^ and the sound wave velocity was 6300 m/s. The two cracks, denoted F1 and F2, were machined using wire electrical discharge machining. The buried depths of F1 and F2 were 60 mm and 35 mm, respectively, and the sampling frequency was 100 MHz. The ultrasonic testing system and the specimen are shown in Figure 5.

**Figure 6:**
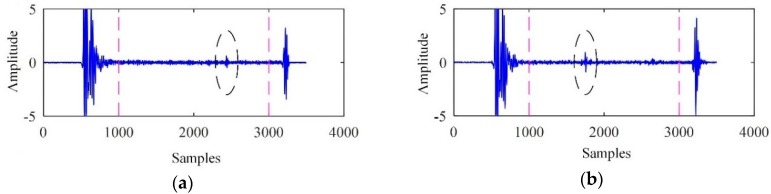
The ultrasonic signals. (**a**) Acquired from F1; (**b**) acquired from F2.

The ultrasonic signals acquired from cracks F1 and F2 are shown in Figure 6. The backscattered signals from instant 1001 to instant 3000 were used for further analysis.

**Figure 7:**
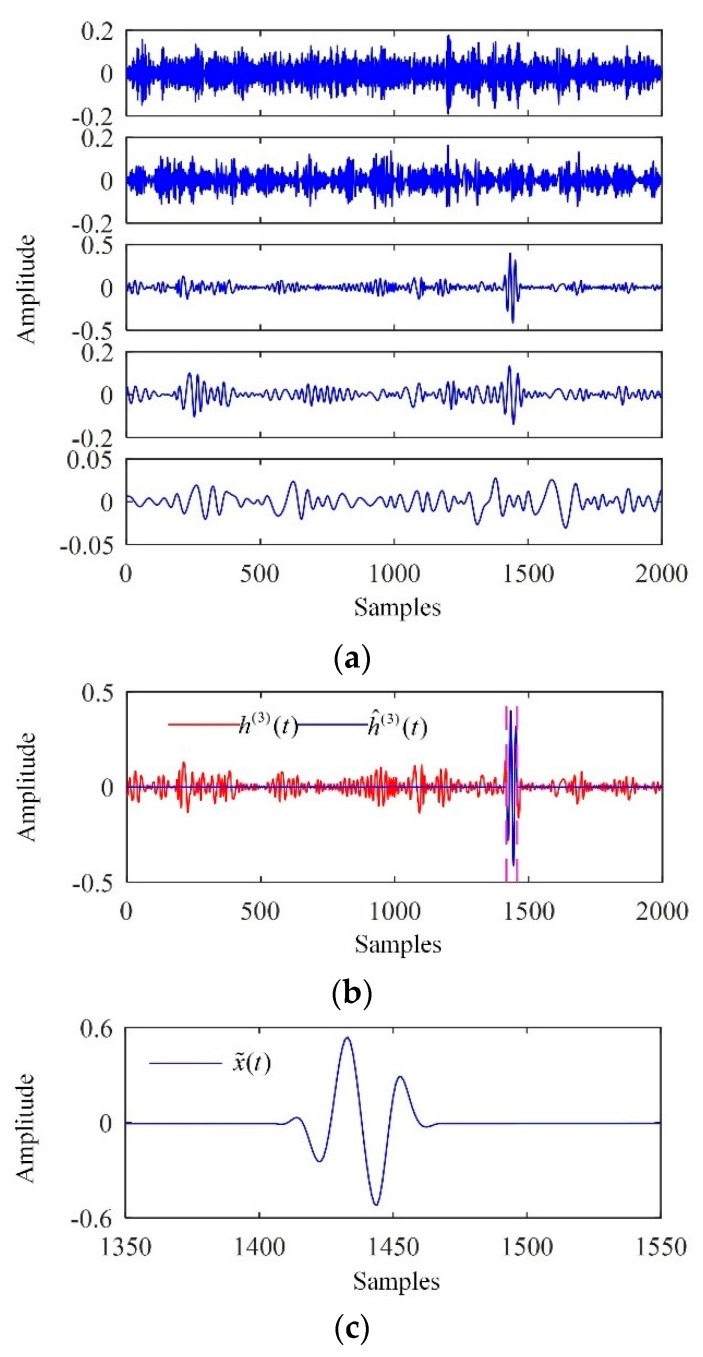
Processing results of the backscattered signal from F1. (**a**) The IMFs extracted; (**b**) the interval in which the flaw echo was located; (**c**) the denoised signal.

The processing results of the backscattered signal from crack F1 are shown in Figure 7. The backscattered signal was first decomposed into a collection of IMFs, as shown in Figure 7a. For brevity, only the first five IMFs are shown. Correspondingly, the SampEn values of the first five IMFs were calculated and are listed in Table 3. As the first relevant mode, the third IMF was used to determine the interval in which the flaw echo was located based on Otsu’s method for thresholding and merging of adjacent mode cells, as shown in Figure 7b. The denoised signal is shown in Figure 7c, indicating that both the backscattering noise and the electronic noise were significantly suppressed.

**Figure 8:**
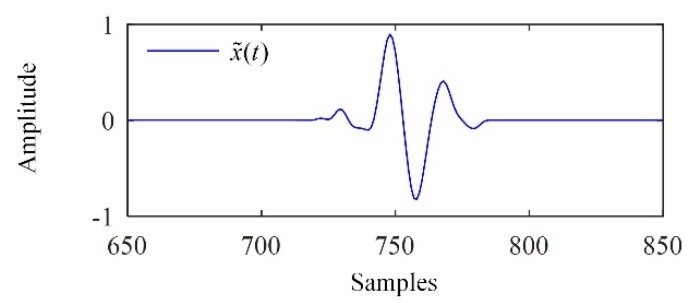
The denoised signal of the ultrasonic signal from F2.

As supplementary information, Figure 8 shows the denoised signal of the backscattered signal from F2, with a significant reduction of the noise.

## 7. Conclusions

An EMD-based methodology was proposed for ultrasonic flaw echo enhancement. The observed signal was decomposed into IMFs by EMD or EEMD. The relevant modes were determined according to the SampEn of the IMFs. Otsu’s method was used for interval thresholding of the first relevant mode, obtaining the interval in which the flaw echo was located. The flaw echoes in all IMFs were separated by the Turkey-Hanning window. The separated flaw echo and the residue were added together, yielding the denoised signal. Simulation results demonstrated that the EMD-based methodology achieved a high SNR and low MCC. Applications of the EMD-based methodology in flaw echo enhancement in experimental signals was also presented.

## Figures and Tables

**Table 1:** SampEn of the IMFs.

*r/SD_u_*	0.1	0.15	0.2
*s*_1_	2.0121	1.7943	1.535
*s*_2_	1.8823	1.4872	1.213
*s*_3_	0.7224	0.6242	0.5682
*s*_4_	0.646	0.5984	0.5616
*s*_5_	0.5501	0.4921	0.4472
*s*_6_	0.4717	0.3589	0.2708
*s*_7_	0.1095	0.0821	0.065
*s*_8_	0.0526	0.036	0.0276

**Table 2:** Performance assessment.

	SNR/dB	MCC/ × 10^−2^
observed	−3.47	1.05
partial reconstruction denoised	1.81 9.42	0.06 0.06

**Table 3:** SampEn of the first five IMFs.

*s*_1_	*s*_2_	*s*_3_	*s*_4_	*s*_5_
1.4902	1.1739	0.5684	0.5508	0.4764
